# Screening Physical Activity in Family Practice: Validity of the Spanish Version of a Brief Physical Activity Questionnaire

**DOI:** 10.1371/journal.pone.0136870

**Published:** 2015-09-17

**Authors:** Anna Puig-Ribera, Carlos Martín-Cantera, Elisa Puigdomenech, Jordi Real, Montserrat Romaguera, José Félix Magdalena-Belio, Jose Ignacio Recio-Rodríguez, Beatriz Rodriguez-Martin, Maria Soledad Arietaleanizbeaskoa, Irene Repiso–Gento, Luis Garcia-Ortiz, EVIDENT Group

**Affiliations:** 1 Sport and Physical Activity Research Group (GREAF), Department of Physical Activity Sciences, Centre for Health and Social Care Research (CEES), University of Vic-Central University of Catalonia (UVic-UCC), Barcelona, Spain; 2 Department of Medicine, Universitat Autònoma de Barcelona, Passeig de Sant Joan Health Center, Catalan Health Service, Barcelona, Spain; 3 Institut Universitari d’Investigació en Atenció Primària Jordi Gol (IDIAP Jordi Gol), Barcelona, Spain; 4 School of Medicine and Health Sciences, Universitat Internacional de Catalunya, Sant Cugat del Valles, Spain; 5 Ca N’Oriac Health Centre. Catalan, Health Service, Spain; 6 Torre Ramona Health Center, Aragón Health Service, Zaragoza, Spain; 7 Primary Care Research Unit, The Alamedilla Health Center, Castilla and Leon Health Service–SACYL, Salamanca, Spain; 8 Occupational Therapy, Logopedia and Nursing Faculty, University of Castilla-La Mancha, Spain; 9 Primary Care Research Unit of Bizkaia, Basque Health Care Service-Osakidetza, Bilbao, Spain; 10 Primary care research unit of Valladolid, Castilla y León Health Service, Valladolid, Spain; 11 Primary Care Research Unit, The Alamedilla Health Center, Castilla and Leon Health Service–SACYL, IBSAL, and Department of Medicine, University of Salamanca, Salamanca, Spain; Texas Tech University Health Science Centers, UNITED STATES

## Abstract

**Objectives:**

The use of brief screening tools to identify inactive patients is essential to improve the efficiency of primary care-based physical activity (PA) programs. However, the current employment of short PA questionnaires within the Spanish primary care pathway is unclear. This study evaluated the validity of the Spanish version of a Brief Physical Activity Assessment Tool (SBPAAT).

**Methods:**

A validation study was carried out within the EVIDENT project. A convenience sample of patients (n = 1,184; age 58.9±13.7 years; 60.5% female) completed the SBPAAT and the 7-day Physical Activity Recall (7DPAR) and, in addition, wore an accelerometer (ActiGraph GT3X) for seven consecutive days. Validity was evaluated by measuring agreement, Kappa correlation coefficients, sensitivity and specificity in achieving current PA recommendations with the 7DPAR. Pearson correlation coefficients with the number of daily minutes engaged in moderate and vigorous intensity PA according to the accelerometer were also assessed. Comparison with accelerometer counts, daily minutes engaged in sedentary, light, moderate, and vigorous intensity PA, total daily kilocalories, and total PA and leisure time expenditure (METs-hour-week) between the sufficiently and insufficiently active groups identified by SBPAAT were reported.

**Results:**

The SBPAAT identified 41.3% sufficiently active (n = 489) and 58.7% insufficiently active (n = 695) patients; it showed moderate validity (k = 0.454, 95% CI: 0.402–0.505) and a specificity and sensitivity of 74.3% and 74.6%, respectively. Validity was fair for identifying daily minutes engaged in moderate (r = 0.215, 95% CI:0.156 to 0.272) and vigorous PA (r = 0.282, 95% CI:0.165 to 0.391). Insufficiently active patients according to the SBPAAT significantly reported fewer counts/minute (-22%), fewer minutes/day of moderate (-11.38) and vigorous PA (-2.69), spent fewer total kilocalories/day (-753), and reported a lower energy cost (METs-hour-week) of physical activities globally (-26.82) and during leisure time (-19.62).

**Conclusions:**

The SBPAAT is a valid tool to identify Spanish-speaking patients who are insufficiently active to achieve health benefits.

## Introduction

Encouraging adults to reach the healthy recommendations for moderate and vigorous physical activity (PA) (150 minutes of moderate or 75 minutes of vigorous intensity PA throughout the week) is a public health priority for chronic disease prevention and management [1;2]. Primary care is a key setting to deliver effective PA interventions [3] with brief PA advice showing an incremental cost-effectiveness ratio of 1,730 Sterling pounds compared with usual care (specified in terms of the probability of patients moving from an inactive to an active state one year later) [4]. Nonetheless, putting these primary care-based interventions into practice does not always take place as desired [5].

The delivery of PA promotion in family medical practice is influenced by many issues such as work overload and shortage of time [4,5]. Consequently, effective intervention strategies need to prioritize time and effort by targeting those patients who can benefit the most: physically inactive and within the preparation stage of change [6]. Using screening tools that fit within the work routine of the primary care setting (short, validated PA questionnaires) can contribute to identify patients with the highest probability of achieving PA at long term [4]. However, current use of brief, validated PA questionnaires within primary care is unclear [4].

Brief PA questionnaires have shown moderate correlation with longer ones and weaker correlations with objective PA measures [7]. Only a few, however, have been validated against objective measures such as accelerometers [8–11]. Moreover, in contrast to similar questionnaires for Italian, German, and French populations there is a scarcity for Spanish-speaking ones [11]. Indeed, while Spanish primary care practitioners use a routine data collection system to record and monitor patient´s PA behavior, no validated short PA questionnaires exist to determine whether Spanish patients are sufficiently active [12]. Providing a validated short PA screening tool to Spanish primary health care practitioners would contribute to appropriately identify patients who are not meeting the current PA guidelines to who discuss PA with [4].

A preliminary study linguistically adapted and validated two brief PA questionnaires [4,8] against the Spanish IPAQ-short version in a small sample of 48 patients [13]. The Spanish version of the Brief Physical Activity Assessment Tool (SBPAAT) [8] reported moderate validity (k = 0.64, 95% IC: 0.50–0.81) and test-retest reliability (k = 0.70, 95% IC:0.53–0.82) [13]. Our study elaborates on previous work with the aim of determining the validity of the SBPAAT [13] using objective PA measures in a diverse sample (gender and age groups) of more than 1,000 Spanish patients.

## Materials and Methods

### Sample

This validation study was carried out within the EVIDENT project. Methods and study population of the EVIDENT study have been previously described in detail [14]. Briefly, this is a multicenter, cross sectional study conducted in six regions of Spain to assess the relationship of PA and dietary patterns with the circadian pattern of blood pressure, arterial stiffness, and endothelial function [14].

From 2011–2012, sixty general practitioners from 6 primary care centers recruited patients aged 20–80 years old (n = 1,553). Exclusion criteria were known coronary or cerebrovascular atherosclerotic disease, heart failure, moderate or severe chronic obstructive pulmonary disease, walking-limiting musculoskeletal disease, advanced respiratory, renal or hepatic disease, severe mental diseases (schizophrenia, acute psychosis or obsessive compulsive disorder), treated oncological disease, pregnancy, terminal illness, and having relative or absolute contraindications for PA practice. Of the 1,553 subjects, three hundred and sixty-nine patients (23.7%) were excluded for not reporting complete information on PA measurements. The final sample size was 1,184 subjects. No statistically significant differences regarding age (p = 0.788), sex (p = 0.842) nor body mass index (p = 0.524) were observed between the 1184 subjects of the present study and the whole sample of the Evident study. The study was approved by an independent ethics committee of Salamanca University Hospital (Spain). All patients signed written informed consent before taking part in the study.

### Study design

Validity of the SBPAAT was evaluated by measuring agreement with the 7 day Physical Activity Recall (7DPAR) in achieving current PA recommendations for health (sufficiently active or meeting PA recommendations). Scores on the SBPAAT were classified into sufficiently and insufficiently active patients by gender and different age groups (<40 years old; 40–64 years old; >65 years old) [15]. Both questionnaires were carried out in an individual face-to-face interview.

Validity of the SBPAAT was also evaluated by comparison with accelerometer activity. Accelerometer measurements were used to (i) assess how well the SBPAAT discriminated on counts per minute, daily minutes engaged in sedentary, light, moderate, and vigorous intensity PA, total daily kilocalories, and total PA expenditure (METs-hour-week) and PA at leisure time (METs-hour-week) between the sufficiently and insufficiently active; (ii) correlate SBPAAT scores evaluating daily minutes of moderate and vigorous PA with the objective measurements of moderate and vigorous PA provided by accelerometers. Accelerometry data was collected within two months following the initial individual interview. The average daily time engaged (minutes) in PA at different intensities was considered an appropriate measure to test the criterion validity of the SBPAAT [15].

### Variables and measurements

A detailed description of variables and measurements has been published elsewhere [14]. Briefly, a trained nurse from each health care center interviewed patients individually, collected data on their PA behavior, and recorded anthropometric data and clinical measurements.

#### Physical activity

Physical activity was measured by the 7DPAR, the SBPAAT, and accelerometers (ActiGraph GT3X). The 7DPAR is a common measure of PA, it has been recognized as a valid and reliable tool and is widely used in epidemiological, clinical, and behavioral change studies [15]. The 7DPAR has shown good validity coefficients against accelerometer data for PA expenditure (r = 0.65; 95% CI: 0.54–0.73) and for time spent on moderate (r = 0.61; 95% CI: 0.50–0.70) and vigorous PA (r = 0.75; 95% CI: 0.67–0.81) in the Spanish population [16]. It is a semi-structured interview (10–15 minutes) that provides a self-estimated number of hours dedicated to physical or occupational activities requiring at least moderate effort in the previous seven days. The categories are: moderate, vigorous, and very vigorous PA. The amount of time spent on each activity is then multiplied by the mean metabolic equivalents (METs) of each category: light activity 1.5 METs, moderate 4 METs, vigorous 6 METs, and very vigorous 10 METs. The sum of the product of time spent in each activity and its estimated mean energy expenditure (MET) provides an estimation of the kilocalories used per day (kcal*kg-1*d-1). Physical activity expenditure is estimated in METs-hour-week. Those individuals doing at least 30 minutes of moderate activity, five days a week, or at least 20 minutes of vigorous activity, 3 days a week, are considered to be sufficiently active. Those who did not reach this level of PA were deemed to be insufficiently active [17].

The Brief Physical Activity Assessment Tool (BPAAT) is a two-item questionnaire administered by health care professionals which measures the frequency and duration of moderate and vigorous PA in an individual’s usual week [8]. By combining the results of both questions (scores can range from 0 to 8) the subject can be classified as sufficiently (≥4 score) or insufficiently active (0–3 score). Subjects are classified as sufficiently active if they report three or more 20 minute sessions of vigorous intensity PA a week; five or more 30 minute sessions of moderate intensity physical activity (including walking) a week; or five or more sessions of any combination of moderate and vigorous intensity PA [8]. Those individuals who do not meet these criteria are considered to be insufficiently active and not meeting current PA recommendations for health [8]. This questionnaire has been preliminary validated for use in the Spanish primary care setting [13].

ActiGraph GT3X accelerometers (ActiGraph, Shalimar, FL, USA) were used to measure PA objectively, which have been previously validated [18–20]. ActiGraph is a monitor that uses a piezoelectric acceleration sensor to filter and convert the signals produced from the sensor in samples collected at a preset frequency in hertz. The samples are summed over a user-specified time sampling interval, called an “epoch”. Output from the ActiGraph is in the form of activity “counts”, where one count is equivalent to 16 milli-g per second, and where g is equal to 9.825 ms^-2^, the acceleration of gravity). Activity “counts” are recorded to the internal memory of accelerometers by converting acceleration units over a given epoch [21]. Individuals wore the accelerometer fastened with an elastic strap to the right side of the waist for seven consecutive days. All subjects received verbal instructions from a trained nurse on how to use the accelerometer. Data were recorded at 1-minute intervals. Sequences of 10 or more consecutive zero counts were considered non-wearing time and were excluded from the analyses. Inclusion criteria consisted of a minimum of four days of recording, including at least one weekend day and at least 600 registered minutes per weekday. The main outcome variable from the activity monitor was the average intensity of PA (counts/minute), calculated with equal weighting given to each day (regardless of registered time per day). PA intensity was classified with the following cut-off points: sedentary (<100 counts/minute), light (100 to 1,952 counts/minute), moderate (1,952 to 5,724 counts/minute), heavy (>5,724 counts/minute), and very heavy (>9,498 counts/minute) [22]. Light, moderate, and vigorous PA were defined as any activity accumulated from all sessions lasting at least 1 min.

#### Other variables

Height, weight, body fat percentage [14], age, sex, occupation, smoking status, alcohol consumption, medical history of cardiovascular diseases, personal history of risk factors (diabetes, arterial hypertension, and dyslipidaemia), drug and usual dietary intake [23] were also measured.

### Statistical analysis

Data on key outcome variables were described using frequencies (percentage) and means (standard deviation). Chi square and Student T statistical tests were used to test differences between the sufficiently and insufficiently active groups identified by the SBPAAT in demographic, lifestyle, and health status variables. Validity of the SBPAAT was evaluated by measuring (i) the percentage of agreement for classifying sufficiently/insufficiently active patients with the 7DPAR, (ii) Kappa coefficient to evaluate how well the SBPAAT discriminated sufficiently and insufficiently active patients as compared to the 7DPAR, and (iii) the ability of the SBPAAT to correctly identify sufficiently active patients (%; sensitivity) and insufficiently active ones (%; specificity).

Validity was also evaluated with (i) Pearson correlation coefficient (r) to assess how well time spent (minutes/day) in moderate (question n° 2) and vigorous PA intensity (question n° 2), as measured by the SBPAAT, correlated with the accelerometer-determined activities of time spent in moderate PA and heavy or very heavy PA (minutes/day; vigorous), and (ii) Mann-Whitney Test to compare differences between the sufficiently and insufficiently active groups on accelerometer daily counts, daily minutes engaged in sedentary, light, moderate, and vigorous intensity PA, total daily kilocalories, total PA expenditure (METs-hour-week), and PA dose at leisure time (METs-hour-week). Data were analyzed using the Statistical Package for the Social Sciences version 15 (SPSS, Chicago, IL, USA). A value of p < 0.05 was considered statistically significant.

## Results

A total of 1,184 subjects were included in the study (58.9±13.7 years of age; 60.5% women). Mean body mass index (BMI) and a percentage of body fat were 27.1 ±4.6 kg/m2 and 34.7±7.6, respectively. Twenty-one percent (n = 252) were current smokers (11.16±9.5 cigarettes/day) and the average weekly alcohol consumption was 4.5±7.8 basic units of alcohol. The most frequent cardiovascular diseases were hypercholesterolemia (30.1%) and diabetes (7.7%). More than 30% followed the Mediterranean Diet and they spent an average of 20.0 ±14.9 hours per week watching television (Table 1).

**Table 1:** Main characteristics of participants in the EVIDENT study by level of physical activity according to the Spanish version of the Brief Physical Activity Assessment Tool (SBPAAT).

		Total		Sufficiently active		Insufficiently active		P-value
All participants	n (%)	1184		489	(41.3)	695	(58.7)	
Sex								
	Men: n (%)	468	(39.5)	232	(47.4)	236	(34.0)	<0.001
	Women: n (%)	716	(60.5)	257	(52.6)	459	(66.0)	
Age								
	Mean: ±SD	58.9	±13.7	55.2	±13.7	54.6	±13.7	0.437
Tobacco consumption								
	No n (%)	561	(47.4)	229	(46.8)	332	(47.8)	<0.001
	Ex-smoker n (%)	371	(31.3)	182	(37.2)	189	(27.2)	
	Currents smoker n (%)	252	(21.3)	78	(16.0)	174	(25.0)	
Cig/week								
	Mean: ±SD	11.2	±9.5	9.1	±7.8	12.1	±10.0	0.020
Alcohol consumption								
	(BUA/week). Mean: ±SD	4.5	± 7.8	4.9	±7.6	4.2	±7.9	0.141
Others variables								
	Ischaemic cardiopathy n (%)	23.0	(2.0)	11	(2.3)	12	(1.7)	0.510
	Cerebrovascular diseases n(%)	17	(1.4)	7	(1.4)	10	(1.4)	0.997
	Heart failure	21	(1.8)	11	(2.3)	10	(1.4)	0.284
	Hypercholesterolemia n(%)	355	(30.1)	143	(29.5)	212	(30.5)	0.712
	Type 2 Diabetes n(%)	91	(7.7)	37	(7.6)	54	(7.8)	0.923
	Compliance with Mediterranean Diet n(%)	397	(33.5)	181	(45.6)	216	(54.4)	0.033
BMI.								
	Mean: ±SD	27.1	±4.6	26.7	±3.9	27.4	±4.9	0.009
% Body fat								
	Mean: ±SD	34.7	±7.6	33.4	±7.4	35.7	±7.5	<0.001
Hours of TV watched/week								
	Mean: ±SD	20.0	±14.9	17.5	±12.6	21.8	±16.1	<0.001

SBPAAT: Spanish version of the Brief Physical Activity Assessment Tool.

Sufficiently active: Individuals who report three or more 20 minute sessions of vigorous intensity PA a week; five or more 30 minute sessions of moderate intensity physical activity (including walking) a week; or five or more sessions of any combination of moderate and vigorous intensity PA.

Insufficiently active: Individuals who do not meet the criteria to be considered sufficiently active.

Cig.: Cigarettes; BUA: Basic unit of alcohol; BMI: Body Mass Index.

SD: Standard deviation.

P-value derived from the Chi-square test and t-student in categorical and continuous variables, respectively.

The SBPAAT identified 41.3% sufficiently active (n = 489) and 58.7% insufficiently active patients (n = 695). No differences between groups were found regarding age, alcohol consumption, and presence of cardiovascular pathology (ischaemic cardiopathy and heart failure), cerebrovascular diseases and chronic conditions (Type 2 diabetes and hypercholesterolemia) (Table 1). In contrast, a higher percentage of women (*p*<0.001), current smokers (*p*<0.001), and those with a greater tobacco consumption (*p* = 0.020) were identified as insufficiently active (66% vs. 52.6%). The mean BMI (p = 0.009), percentage of body fat (<0.001), and weekly hours watching television (p<0.001) were all higher among insufficiently active individuals (Table 1).

### Validity data

**Fig 1:**
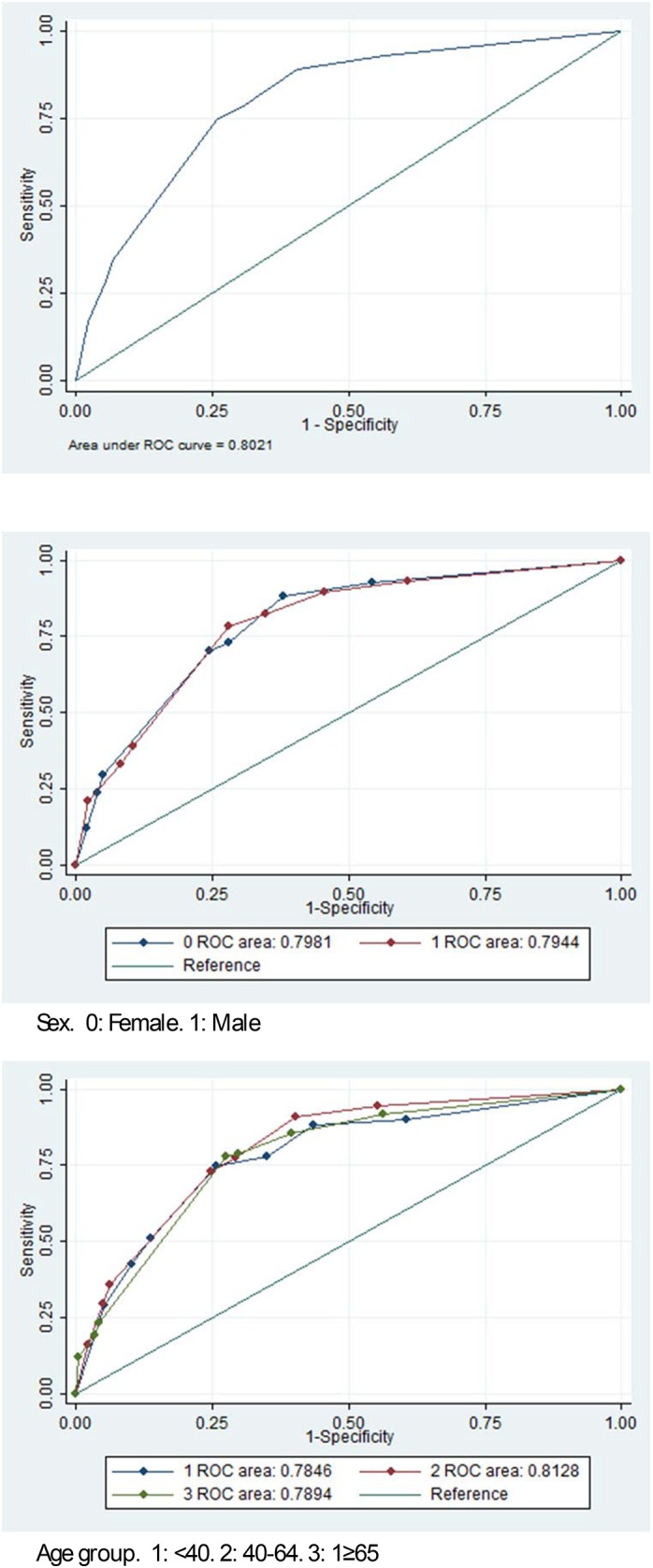
ROC curves for the participants in the EVIDENT study group and according to sex and age groups.

Table 2 shows the percentage of agreement for identifying sufficiently/insufficiently active patients between the SBPAAT and the 7DPAR. Eighty-six percent of insufficiently active participants (according to the SBPAAT) were classified as insufficiently active by the 7DPAR (negative predictive value), whilst from those considered as sufficiently active by the SBPAAT, 57.7% were also considered sufficiently active by the 7DPAR (positive predictive value). Negative predictive values were higher among women (88.5%) and among older individuals (87.7%). Positive predictive values were higher among men (67.7%) and younger individuals (59.5%). The specificity and sensibility of the SBPAAT were 0.74 (95% CI:0.713 to 0.773) and 0.75 (95% CI:0.702 to 0.790), respectively. Specificity and sensibility values were quite similar regarding sex and age groups (Table 2). The global Kappa Index was 0.454 (CI95%: 0.405–0.505) and ranged between 0.4 and 0.5 depending on the demographic group (Table 2). Fig 1 shows the global ROC curve with an area under the curve of 0.802 (CI95%: 0.78–0.83). ROC curve areas according to sex and group age were similar to the total one (Fig 1).

**Table 2:** Comparison of individuals classified as being sufficiently and insufficiently active according to both tests (7DPAR and SBPAAT) by age and gender.

		SBPAAT									
		Insufficiently Active^1^ (n = 695)		Sufficiently Active^2^ (n = 489)		Specificity no active		Sensibility Active		Global value	Kappa Index
7DPAR		N	(%)	N	(%)	%	(CI 95%)	%	(CI 95%)	%	
	**Total**										
	Sufficiently active^a^	96	(13.8)	282	(57.7)	-	-	0.75	(0.702–0.790)	0.74	0.454
	Insufficiently active^b^	599	(86.2)	207	(42.3)	0.74	(0.713–0.773)	-	-		
	**Sex**										
	Men										
	Sufficiently active	43	(18.2)	157	(67.7)	-	-	0.79	(0.728–0.842)	0.75	0.495
	Insufficiently active	193	(81.8)	75	(32.3)	0.72	(0.666–0.774)	-	-		
	Women										
	Sufficiently active	53	(11.5)	125	(48.6)	-	-	0.70	(0.635–0.769)	0.74	0.398
	Insufficiently active	406	(88.5)	132	(51.4)	0.75	(0.718–0.791)	-	-		
	**Age (years old)**										
	< 40										
	Sufficiently active	15	(14.7)	44	(59.5)	-	-	0.75	(0.635–0.857)	0.74	0.460
	Insufficiently active	87	(85.3)	30	(40.5)	0.74	(0.664–0.823)	-	-		
	40–65										
	Sufficiently active	57	(14.3)	153	(57.7)	-	-	0.73	(0.668–0.789)	0.75	0.450
	Insufficiently active	341	(85.7)	112	(42.3)	0.75	(0.713–0.792)				
	≥65										
	Sufficiently active	24	(12.3)	85	(56.7)	-	-	0.78	(0.702–0.858)	0.74	0.458
	Insufficiently active	171	(87.7)	65	(43.3)	0.72	(0.668–0.782)	-	-		

SBPAAT: Spanish version of the Brief Physical Activity Assessment Tool; 7DPAR: 7-day Physical Activity Recall; CI 95%: Confidence Interval 95%

^1^Insufficiently active: Individuals who do not report three or more 20 minute sessions of vigorous intensity PA a week; five or more 30 minute sessions of moderate intensity physical activity (including walking) a week; or five or more sessions of any combination of moderate and vigorous intensity PA.

^2^Sufficiently active: Individuals who report three or more 20 minute sessions of vigorous intensity PA a week; five or more 30 minute sessions of moderate intensity physical activity (including walking) a week; or five or more sessions of any combination of moderate and vigorous intensity PA.

^a^Insufficiently active: Individuals not doing at least 30 minutes of moderate activity, five days a week, or at least 20 minutes of vigorous activity, 3 days a week.

^b^Sufficiently active: Those individuals doing at least 30 minutes of moderate activity, five days a week, or at least 20 minutes of vigorous activity, 3 days a week.

**Fig 2:**
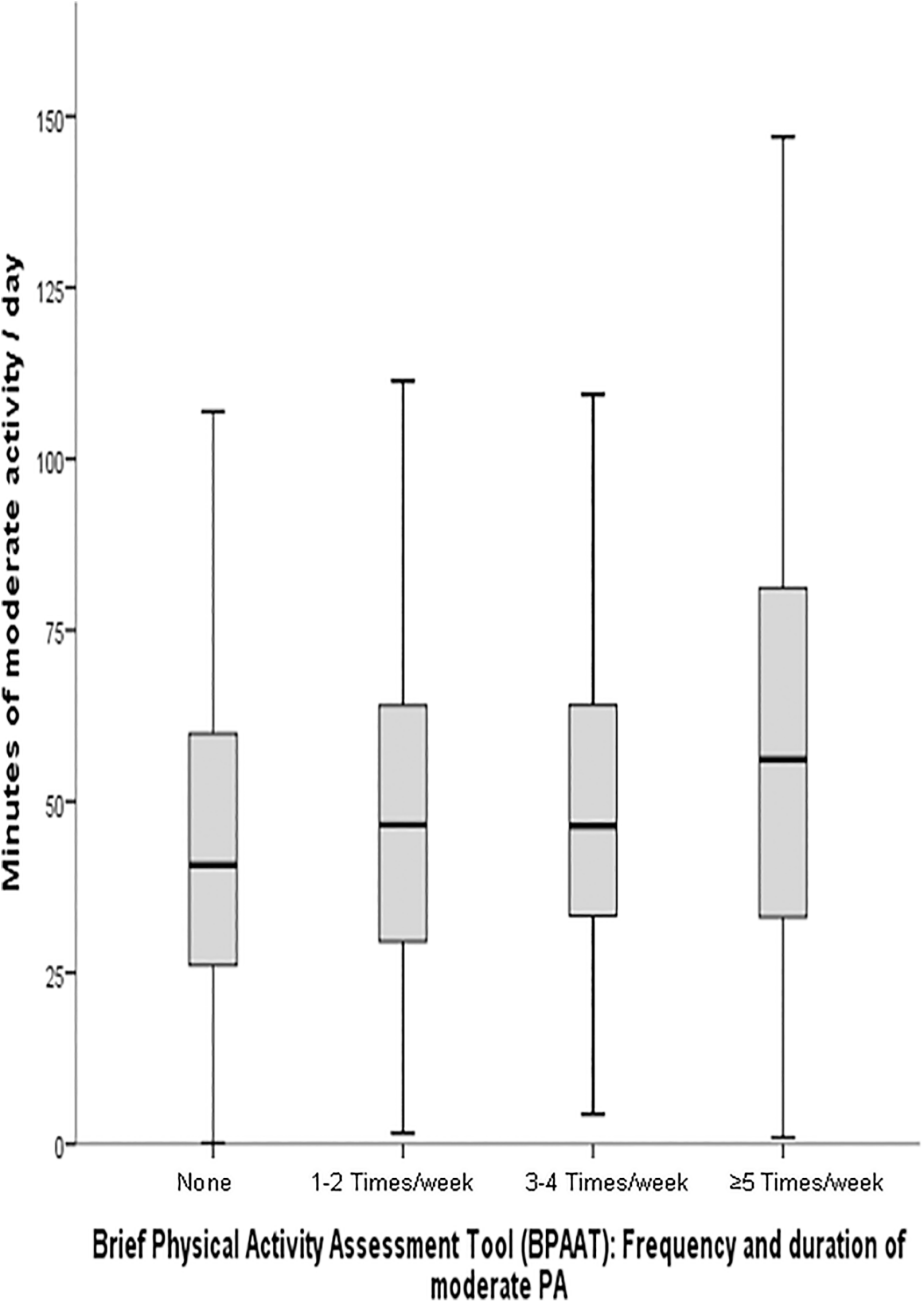
Box-plot of minutes of moderate activity/day according to the brief physical activity assessment tool (BPAAT Test).

Table 3 shows the differences in accelerometer-determined activities between the sufficiently and the insufficiently active groups identified by the SBPAAT. Mean counts per minute (+52 counts/minute; +22%), moderate (+11.88 minutes/day) to vigorous PA (+2.68 minutes/day), kilocalories spent a day (+752.81 kilocalories/day), and METs-hour-week (+26.82) were higher in the sufficiently active group than the insufficiently active one (*p*<0.001). Light PA did not show significant differences between groups. A similar pattern was identified for different age groups (Table 4) and gender (Table 5) with the exception of mean minutes of light activity that were higher among sufficiently active women (349.3 minutes/day vs. 339.3 minutes/day). Validity for identifying daily minutes engaged in moderate and vigorous PA was r = 0.215 (95% CI 0.156 to 0.272) and r = 0.282 (95% CI: 0.165 to 0.391), respectively (Fig 2).

**Table 3:** Comparison of accelerometer-determined activities between the sufficiently and insufficiently active groups labelled by the SBPAAT.

	SBPAAT										
	Sufficiently active					Insufficiently active					P-value
	Mean	SD	P25	P50	P75	Mean	SD	P25	P50	P75	
Counts/minute	286.1	119.46	201.92	267.94	350.38	233.4000	88.25	168.41	221.69	284.18	<0.001
Minutes being sedentary / day	1049.7	88.90	996.88	1055.29	1106.68	1067.47	91.92	1009.9	1073.5	1131.14	0.001
Minutes of light activity / day	330.4	78.89	281.36	329.45	371.61	326.54	86.23	267.57	320.76	373.42	0.208
Minutes of moderate activity / day	58.11	34.35	33.92	53.57	76.07	46.72	26.31	26.87	43.08	61.42	<0.001
Minutes of heavy-very heavy activity / day	3.57	9.53	0.00	0.00	2.11	0.88	3.7	0	0	0.14	<0.001
Total Kcal spend / day	2570.00	1903.25	1147.93	2183.94	3516.46	1817.19	1.32E+03	860.36	1534.3	2410.26	<0.001
METsWeekDose (METS/hour/week)	36.24	54.09	9.00	23.33	45.00	9.41	21.5	0	0.66	10	<0.001
METsWeekDose Leisure time (METS/hour/week)	26.98	29.60	6.00	18.67	37.00	7.35	15.07	0	0	9	<0.001

SBPAAT: Spanish version of the Brief Physical Activity Assessment Tool.

SD: Standard Deviation.

P: percentile.

P-value derived from Mann-Whitney Test.

The accelerometer did not record all physical activities such as aquatic or muscular activities.

**Table 4:** Comparison of accelerometer-determined activities between the sufficiently and insufficiently active (according to SBPAAT) by age groups.

	SBPAAT										
	Sufficiently active					Insufficiently active					P-value
	Mean	SD	P25	P50	P75	Mean	SD	P25	P50	P75	
**<40 years old**											
Counts/minute	271.3000	123.6	189.76	268.55	309.37	207.8700	73.33	153.78	208.41	240.7	<0.001
Minutes being sedentary / day	1058.55	108.48	1004.03	1064.96	1119.4	1089.21	98.49	1043.89	1092.5	1157.9	0.037
Minutes of light activity / day	329.14	92.24	266.71	326.31	394.5	313.29	94.16	241.53	307.08	366.51	0.172
Minutes of moderate activity / day	50.18	31.8	25.04	46.21	64.71	37.98	19.51	23.57	34.78	48.5	0.011
Minutes of heavy-very heavy activity / day	3.9	6.19	0	0.57	4.64	0.68	2.04	0	0	0.42	<0.001
Total Kcal spend / day	2298.8	1.80E+03	915.99	2080.9	3098.1	1390.02	1.14E+03	674.55	979.58	1807.12	<0.001
METsWeekDose (METS/hour/week)	36.93	40.68	11	24.5	45.91	10.48	21.1	0	0.66	12	<0.001
METsWeekDose Leisure time (METS/hour/week)	32.2	34.22	10.5	20	40.75	6.51	13.79	0	0	8.25	<0.001
**40–64 years old**											
Counts/minute	1303.70	115.43	229.83	296.62	367.09	249.07	90.27	183.35	239.92	306.97	<0.001
Minutes being sedentary / day	1033.51	85.01	979.86	1037.97	1085.39	1049.82	89.68	994.05	1055.35	1112.21	0.017
Minutes of light activity / day	343.65	77.76	295.66	344.68	384.50	342.19	83.39	288.22	338.88	395.93	0.631
Minutes of moderate activity / day	59.86	28.70	39.09	57.29	75.21	48.67	26.48	27.82	44.21	63.00	<0.001
Minutes of heavy-very heavy activity / day	4.91	11.92	0.00	0.15	2.79	1.09	3.98	0.00	0.00	0.29	<0.001
Total Kcal spend / day	2725.76	1854.99	1353.59	2365.86	3577.78	1927.89	1353.17	976.13	1670.08	2494.08	<0.001
METsWeekDose (METS/hour/week)	35.49	62.48	9.00	20.00	41.03	9.46	20.69	0.00	0.00	10.67	<0.001
METsWeekDose Leisure time (METS/hour/week)	22.65	22.49	5.92	17.00	32.83	7.99	16.61	0.00	0.00	10.00	<0.001
**≥65 years old**											
Counts/Minute	256.08	117.62	167.46	234.29	328.04	307.21	84.83	151.15	202.21	260.52	0.002
Minutes being sedentary / day	1074.06	78.79	1019.27	1078.29	1127.68	1092.12	84.97	1043.86	1090.14	1156.29	0.037
Minutes of light activity / day	307.62	68.29	259.32	311.78	353.15	301.56	80.82	250.29	297.05	348.57	0.256
Minutes of moderate activity / day	58.94	43.28	25.10	52.00	85.85	47.35	28.22	26.43	45.41	63.09	0.04
Minutes of heavy-very heavy activity / day	1.05	4.16	0.00	0.00	0.00	0.57	3.79	0.00	0.00	0.00	0.106
Total Kcal spend / day	2428.62	2021.11	846.45	1973.16	3563.64	1814.71	1303.70	821.00	1519.33	2497.01	0.023
METsWeekDose (METS/hour/week)	37.21	43.04	6.92	25.83	56.00	8.76	23.36	0.00	0.67	8.33	<0.001
METsWeekDose Leisure time (METS/hour/week)	32.06	36.43	6.00	24.00	40.42	6.50	12.11	0.00	0.00	8.00	<0.001

SBPAAT: Spanish version of the Brief Physical Activity Assessment Tool; SD: Standard Deviation P: percentile;P-value derived from Mann-Whitney Test.

The accelerometer did not record all physical activities such as aquatic or muscular activities.

**Table 5:** Comparison of accelerometer-determined activities between the sufficiently and insufficiently active (according to SBPAAT) by gender.

	SBPAAT										
	Sufficiently active					Insufficiently active					P-value
	Mean	SD	P25	P50	P75	Mean	SD	P25	P50	P75	
**Women**											
Counts/minute	276.53	108.2	199.56	263.99	339.1	225.92	82.54	164.96	216.13	273.34	<0.001
Minutes being sedentary / day	1036.02	88.15	980.14	1041.57	1085.88	1058.7	91.6	1000.9	1067.28	1122.65	<0.001
Minutes of light activity / day	349.3	73.85	309.07	351.57	389.02	339.33	85.5	278.71	338.85	386.74	0.024
Minutes of moderate activity / day	53.94	30.59	31.42	49.58	71.06	43.07	24.18	25.15	39.85	57.6644	<0.001
Minutes of heavy-very heavy activity / day	2.35	6.33	0	0	0.99	0.46	1.76	0	0	0.14	<0.001
Total Kcal spend / day	1979.56	1.40E+03	934.64	1563.5	2719.16	1426.35	9.74E+02	722.57	1263.86	1916.22	<0.001
METsWeekDose (METS/hour/week)	32.61	64.43	2.83	17	38.66	8.04	17.8	0	0	9.33	<0.001
METsWeekDose Leisure time (METS/hour/week)	20.84	27.44	0.73	13.33	28	6.74	15.21	0	0	8	<0.001
**Men**	247.0000	130.21	203.15	279.62	356.64	247.9600	96.96	180.16	239.05	304.04	<0.001
Counts/minute	1064.94	87.4	1016.2	1074.25	1122.85	1084.52	90.31	1032.1	1087.21	1150.1	0.026
Minutes being sedentary / day	309.46	79.14	252.56	308.96	349.14	301.67	82.28	245.37	299.17	343.98	0.381
Minutes of light activity / day	62.72	37.61	36.717	56.64	82.43	53.83	28.78	31.364	49.14	71.35	0.014
Minutes of moderate activity / day	4.92	11.98	0	0.14	3.82	1.69	5.79	0	0	0.42	<0.001
Minutes of heavy-very heavy activity / day	3224.06	2.16E+03	1756.1	2837.84	4160.65	2577.35	1.56E+03	1425.7	2339.51	3399.65	0.001
Total Kcal spend / day	40.24	39.34	15	30	52	12.07	27.16	0	2.5	14.12	<0.001
METsWeekDose (METS/hour/week)	33.77	30.47	12	27.5	42.5	8.54	14.74	0	1.33	11.66	<0.001
METsWeekDose Leisure time (METS/hour/week)	276.53	108.2	199.56	263.99	339.1	225.92	82.54	164.96	216.13	273.34	<0.001

SBPAAT: Spanish version of the Brief Physical Activity Assessment Tool.

SD: Standard Deviation P: percentile.

P-value derived from Mann-Whitney Test.

The accelerometer did not record all physical activities such as aquatic or muscular activities.

## Discussion

Our study evaluated validity of the Spanish version of the Brief Physical Activity Assessment Tool (SBPAAT) [8] in a diverse sample of more than 1,000 patients. To the best of our knowledge, this study provides for the first time a valid short assessment tool to identify Spanish-speaking patients in need of PA interventions. This will support primary care practitioners to advise adults who are insufficiently active to do more PA without relying on visual cues to asses PA levels (for example body weight) [4].

The main result of this study highlighted the fact that the SBPAAT had moderate concurrent validity to determine sufficiently and insufficiently active patients, showing an acceptable kappa correlation coefficient in terms of self-reported PA assessment (kappa coefficient = 0.454) [24]. These results indicate that the SBPAAT performed at least as well as the original questionnaire for English speakers (kappa coefficient = 0.467) in a sample of 509 Australian patients [9]. The preliminary validation study of the SBPAAT reported a higher kappa correlation coefficient [13]. Nevertheless, it should be noted that SBPPAT scores were compared with the Spanish version of the IPAQ-short form [25] which has shown weaker correlations in measuring time spent doing vigorous and total PA than the Spanish version of the 7DPAR (0.38 vs. 0.75; 0.27 vs. 0.65 respectively) [16].

Our results suggest that validity of the SBPAAT is as good as longer self-reported PA questionnaires [15]. A total of 130 PA questionnaires were examined by a systematic review reporting objective criterion-related validity data. The correlation coefficient of the SBPAAT identifying sufficiently versus insufficiently active patients fits within the range of correlations coefficients identified for the existing (Pearson r = 0.34–085; Spearman r = 0.21–0.60) and new PA questionnaires (Pearson r = 0.20–0.63; Spearman r = 0.23–0.74) (15). The SBPAAT also performed as well as other brief PA questionnaires. Ball et al (2014) tested the validity of the Physical Activity Vital Sign (PAVS) against accelerometry (n = 45). In a similar manner to the SBPAAT, the PAVS showed moderate correlations for identifying sufficiently/insufficiently active individuals (k = 0.46, p<0.001). Milton et al (2013) assessed the validity of a single-item PA self-reported tool against accelerometry (n = 66). Correlation coefficients (k = 0.23, 95% CI 0.05 to 0.41) [10] in classifying participants as sufficiently/insufficiently active were weaker than the SBPAAT. However, when the single-item PA questionnaire was validated against the Global Physical Activity Questionnaire [7], the correlation coefficient (kappa = 0.63, 95% CI 0.54 to 0.72) was higher. Mader et al (2006) tested three brief PA questionnaires against accelerometer records [26]. A moderate relationship for continuous questionnaires was observed which was somewhat lower for dichotomous data; moreover, the authors concluded that vigorous activity was overestimated in all the questionnaires studied.

Only a few studies have assessed the validity properties of brief PA questionnaires by gender and age groups. In a similar manner to SBPAAT, Wanner et al (2014) reported that a single item PA questionnaire performed better at identifying sufficiently/insufficiently active patients in women and younger people (<40 years old) [11]. In patients >65 years old, our study reported that SBPAAT performed better at identifying the sufficiently active rather than insufficiently active (k = 0.458) while Gill et al (2012) identified a weaker correlation coefficient for a single PA question (r = 0.28 to 0.57) in a sample of older adults. Our study contributes to the few validation studies that have evaluated short PA questionnaires across age groups [15].

Recently, two brief PA questionnaires (Stanford Brief Activity Survey, SBAS; the Rapid Assessment of Physical Activity-RAPA) were validated for Spanish-speaking Mexicans residing in the United States [27] (n = 34). The SBPAAT showed better specificity and sensitivity than the SBAS (0.47 and 0.60) and was similar to the RAPA (0.73 and 0.75). In Spain, the Spanish version of the short Minnesota Leisure time PA questionnaire was developed to be used in primary care [28]. However, this questionnaire identifies PA at leisure time rather than the achievement of current PA recommendations. The Spanish IPAQ-short version [25] has reported similar values for specificity and sensitivity as the SBPAAT (75% vs.74.3% and 75% vs. 74.6% respectively). However, the weighted Kappa was higher for the SBPAAT (k = 0.454) than for the IPAQ short form (k = 0.33, p<0.05) [24].

The SBPAAT showed fair validity in measuring time spent (minutes/day) doing moderate or vigorous PA against accelerometry. Other short PA questionnaires (RAPA) have reported higher correlation coefficients between questionnaire scores and accelerometry measures of minutes/day doing moderate and vigorous PA (r = 0.38, r = 0.45, respectively) [27]. However, the SBPAAT performed as well as the Spanish IPAQ-short version in identifying daily minutes of vigorous PA (r = 0.27; p<0.05) and performed better in identifying daily minutes of moderate PA [25]. Finally, the SBPAAT could not detect significant differences in light intensity activity between the insufficiently and sufficiently active patients. A brief assessment tool has been recently designed to identify patients with high levels of sedentarism and low daily PA (Rapid Assessment Disuse Index-RADI) [29]. It demonstrates moderate validity in identifying light-intensity PA (p = 0.40; p<0.01) and sedentary time (p = 0.40; p<0.01). This indicates that the RADI might be a more adequate brief assessment tool to assess light-intensity PA than SBPAAT.

There are several limitations to the present study. First, this is a cross-sectional study that has not been able to detect changes in PA over time. Second, participation was voluntary and it is well known that subjects who agree to participate tend to undertake healthier lifestyle behaviors. Third, despite the valuable information gathered by the accelerometer, this device does not record all physical activities such as aquatic or muscular activities. Finally, accelerometry measurements were taken within 2 months after completing the SBPAAT, which means it measured PA levels at different time points.

In summary, the SBPAAT is a valid tool to identify Spanish-speaking patients who are not active enough to gain health benefits and shows acceptable validity across age groups and gender. Using the SBPAAT as a screening tool for PA in the Spanish primary care system could contribute to increasing the efficiency of primary care-based PA programs by providing reliable records on patient´s PA information and identifying those to whom PA promotion programs should be offered.

## Supporting Information

S1 Dataset Data set in SPSS format (sav). (ZIP)
